# Variability in Nurses’ Assessments and Concordance of Multimodal Generative AI Models in Image-Based Wound Assessment

**DOI:** 10.3390/healthcare14213587

**Published:** 2026-10-23

**Authors:** Alberto González-García, Ingrid Villacorta Dos Reis, Cristina Liebana-Presa, Elena Andina-Díaz, David Bermejo-Martínez, Silvia Pérez-González

**Affiliations:** 1 HeQoL Research Group, Department of Nursing and Physiotherapy, Cuidados Enfermeros Para la Calidad de Vida Relacionada con la Salud (CECAVIS), León Biomedical Research Institute (IBIOLEÓN), Universidad de León, 24071 León, Spain; agong@unileon.es (A.G.-G.); cristina.liebana@unileon.es (C.L.-P.); elena.andina@unileon.es (E.A.-D.); 2 Department of Nursing and Physiotherapy, Faculty of Health Sciences, Universidad de León, Campus de Vegazana s/n, 24071 León, Spain; ivilld02@estudiantes.unileon.es; 3 SALBIS Research Group, Department of Nursing and Physiotherapy, Universidad de León, 24071 León, Spain; 4 SALBIS Research Group, Cuidados Enfermeros Para la Calidad de Vida Relacionada con la Salud (CECAVIS), León Biomedical Research Institute (IBIOLEÓN), Hospital de León, Complejo Asistencial Universitario de León, 24071 León, Spain; sperg@unileon.es

**Keywords:** artificial intelligence, clinical decision-making, large language models, nursing assessment, observer variation, wound care, wounds and injuries

## Abstract

Background/Objectives: Image-based wound assessment may vary among experienced nurses. This study examined inter-nurse variability and concordance with three multimodal generative artificial intelligence models. Methods: In this exploratory cross-sectional comparative study, 18 nurses with wound-care expertise and ChatGPT, Gemini, and Claude independently assessed 30 de-identified wound images from León, Spain, across seven assessment and treatment-related domains. Inter-nurse agreement was summarized using modal shares and Gwet’s AC1. Concordance with the nurse modal set required an exact match with any response in the nurse modal set. Overall confidence intervals were estimated by case-clustered bootstrap resampling. No independent clinical reference standard was established. Results: The analyses quantified inter-nurse agreement and model concordance with the nurse modal set, not diagnostic accuracy. Inter-nurse agreement was highest for wound etiology (mean modal share, 71.3%; AC1, 0.520) and lowest for primary dressing selection (43.7%; AC1, 0.163). Overall concordance was 63.3% (95% confidence interval, 56.7–70.0) for Gemini, 48.6% (40.5–56.2) for ChatGPT, and 44.8% (38.6–51.4) for Claude. Gemini exceeded ChatGPT by 14.8 percentage points and Claude by 18.6 percentage points in exploratory comparisons of overall concordance with the nurse modal set. Complete intermodel agreement occurred in 38.6% of 210 case–domain tasks. Conclusions: Image-based wound assessments varied among experienced nurses, and model concordance with the nurse modal set differed across assessment domains and evaluated systems. Concordance with the nurse modal set reflects agreement with the panel’s most frequently selected responses. It does not establish clinical correctness, clinical validity, or an ability to resolve clinical ambiguity: image-only assessment, the small purposive sample, and reliance on a nurse-derived comparator limit interpretation. Further validation in clinical contexts and against independent reference standards is needed before these models can support wound-care decisions.

## 1. Introduction

Wounds are heterogeneous clinical problems whose assessment links etiology, wound-bed tissue, moisture, infection, periwound status, healing trajectory, and patient context to a management plan [1]. Chronic wounds in particular arise from interacting vascular, metabolic, inflammatory, mechanical, and behavioral factors; an image can show some local features but cannot capture pain, odor, perfusion, systemic signs, comorbidity, or treatment history [2]. Competent wound care therefore requires both structured observation and context-sensitive clinical reasoning [3,4].

Variation in wound assessment can affect documentation and treatment planning. Clinicians may classify the same wound tissue differently [5], and researchers have investigated structured decision support to reduce practice variation [6]. Differences in judgment may reflect ambiguous visual information, inconsistent terminology, variation in preparation, or legitimate patient-specific considerations [3,7].

Against this background, emerging technologies such as artificial intelligence offer opportunities to support nurses’ decision-making in wound care [8]. Machine-learning and image-recognition systems can analyze clinically relevant wound characteristics, including wound dimensions, depth, tissue composition, and changes over time [9]. Other artificial intelligence approaches have been developed to identify factors associated with delayed healing or adverse outcomes [10]. By integrating these functions, artificial intelligence could support earlier recognition of complications, more systematic monitoring, and better-informed selection of preventive and therapeutic interventions [11]. However, most of these applications are purpose-built systems developed to perform narrowly defined wound-care tasks and require task-specific training, calibration, and clinical validation [9,10,12].

Widely accessible multimodal generative AI models, including ChatGPT, Gemini, and Claude, can process photographs and natural-language instructions to generate wound assessments or management suggestions [13]. Their conversational interfaces extend the range of potential applications discussed in wound-care reviews [14]. Broader evidence also suggests a potential role for artificial intelligence in supporting nurses’ clinical decisions, although technologies, tasks, and validation approaches vary substantially [15]. Therefore, general-purpose models should be distinguished from dedicated wound-care systems: we cannot assume they can interpret wound characteristics and recommend appropriate management. Performance may depend on the model, visual ambiguity, the available clinical context, and the prompting and response format [13].

Consequently, before general-purpose multimodal generative artificial intelligence models can be considered reliable tools for supporting wound-care decisions, their outputs must be evaluated against expert clinical judgment and the variability already present among clinicians [16]. This distinction is important because disagreement between a model and an individual clinician may reflect model error. However, it may also reflect genuine ambiguity in the visual information or limited agreement among experts [17]. Accordingly, this study addressed the following research question: To what extent do clinical decisions generated by multimodal generative artificial intelligence models converge with or diverge from those made by expert nurses when assessing wound images, and does this level of agreement vary according to the task-level inter-nurse agreement?

### Objectives

The research objectives are as follows:

• To analyze variability in image-based wound assessment and treatment-related decision-making among expert nurses and quantify the concordance of three widely accessible multimodal generative artificial intelligence models with the nurse modal set.
• To quantify inter-nurse agreement across five wound-assessment dimensions: wound etiology, predominant wound-bed tissue, moisture level, signs of infection, and periwound condition.
• To analyze variability in treatment-related decision-making, specifically in determining the primary therapeutic objective and selecting the primary wound dressing.
• To describe variation in the concentration of nurse responses across case–domain tasks using modal shares.
• To evaluate intermodel agreement and each model’s concordance with the nurse modal set across the assessment and treatment-related domains.
• To identify patterns of alignment and disagreement between the models and nurses, and explore whether concordance varied across task-level inter-nurse agreement strata.

## 2. Materials and Methods

### 2.1. Study Design

This exploratory cross-sectional comparative agreement study examined wound assessment and treatment-related decisions made by 18 nurses with wound-care expertise and by three general-purpose multimodal generative artificial intelligence models. Nurses and models independently evaluated the same 30 standardized wound-image cases under image-only conditions. The study characterized inter-nurse agreement, agreement among the models, and each model’s concordance with the nurse modal set. It also explored whether concordance with the nurse modal set varied with task-level inter-nurse agreement. Because the study did not establish an independently adjudicated reference standard, it evaluated response agreement rather than diagnostic accuracy or clinical effectiveness. Reporting was organized with reference to the STROBE statement for cross-sectional studies and GRRAS recommendations for agreement studies [18,19], with reference to applicable MI-CLEAR-LLM recommendations [20]. The study was not preregistered.

### 2.2. Setting and Wound-Image Acquisition

We prospectively obtained clinical photographs from 30 patients receiving care at a university hospital and associated primary care centers in northwestern Spain in May 2026. Each patient contributed one wound. During a scheduled dressing change, the principal investigator removed the existing dressing, gently cleansed the wound and surrounding skin, and photographed the wound before debridement, topical treatment, or application of a new dressing.

Three consecutive photographs were obtained for each wound using the same iPhone 17 Pro (Apple Inc., Cupertino, CA, USA), at an approximate camera-to-wound distance of 50 cm. Photographs were acquired at a resolution of 48 megapixels. We took photographs in landscape orientation, with the camera positioned approximately perpendicular to the wound, a green drape as the background, and natural light without flash. Images captured the wound bed, wound edges, and periwound skin. We applied no filters or image-enhancement procedures.

All research team members selected one photograph per wound, and the majority vote determined the final selection. Selection considered image sharpness, uniformity of illumination, minimal perspective distortion, and complete visualization of the wound and periwound skin. We retained a single photograph for each case to present the same visual information to all nurse and model evaluators. This procedure yielded 30 study images, one per patient. We de-identified selected images and removed identifying metadata before evaluation.

### 2.3. Construction of the Wound-Image Cases

We selected cases through purposive quota sampling from patients encountered during clinical care in May 2026. Five wound categories were included: pressure injuries, venous ulcers, arterial ulcers, diabetic foot ulcers, and surgical wounds healing by secondary intention. Six cases were selected per category, yielding 30 wounds from 30 patients. All 30 patients approached agreed and provided informed consent, yielding a 100% consent rate among those approached. Once a category quota was reached, selection focused on underrepresented categories. These categories guided recruitment and were not used as an independently adjudicated reference standard for evaluating nurse or model responses.

Within the wound-type quotas, the investigators sought variation in visible wound-bed tissue, moisture or exudate, signs compatible with infection, and periwound condition among the available cases. This selection strategy sought to avoid concentrating the dataset on a narrow range of visual presentations; it did not ensure equal frequencies across all visual features or reproduce their prevalence in routine practice. Wounds at anatomical sites where photography could compromise patient anonymity were excluded. Each case included one de-identified photograph with no accompanying demographic or clinical information. The resulting sample covered five wound categories but was not statistically representative of the wider wound-care population.

We set a target of 30 cases before data collection and allocated cases equally across the five wound categories. Image sets of this size have been used in previous wound-image assessment studies, including a comparison of clinicians and an AI system for maceration detection and an evaluation of periwound skin classification [21,22]. These studies provide methodological precedents rather than evidence that 30 cases are sufficient for all agreement outcomes. Accordingly, we conducted an exploratory study and reported uncertainty using confidence intervals. The sample was not intended to support definitive conclusions within individual wound categories or population-level generalization. No formal precision-based or simulation-based sample-size calculation was performed.

### 2.4. Nurse Participants

We purposively recruited registered nurses from hospital, primary care, and long-term care settings. Eligibility required current clinical practice, at least five years of nursing experience, regular involvement in complex-wound assessment and treatment, and formal wound-specific education. We invited 30 eligible nurses, and they initially agreed to participate. Of these, 18 completed assessments of all 30 wound images, yielding a 60% completion rate among those invited. The remaining 12 did not undertake the assessment. We did not solicit reasons for nonparticipation to avoid any perceived pressure to participate. We had sociodemographic and professional characteristics only for the 18 nurses who completed the assessment; we had no such data for the 12 nonparticipants. We applied no additional selection criteria among nurses who initially agreed; the final panel comprised those who completed the assessment rather than a statistically determined target. We defined expertise operationally by clinical experience, current wound-management responsibilities, and wound-specific education rather than professional title alone. Participants reported age, sex, nursing and wound-care experience, principal clinical setting, academic qualifications, and wound-specific education. All participants completed the 30 cases, with no missing participant-level assessments.

### 2.5. Assessment Instrument and Nurse Evaluation

A structured study-specific questionnaire was administered through Google Forms.

The questionnaire’s assessment domains and response categories were developed using GNEAUPP and EWMA guidance and the TIME/TIMERS framework. The questionnaire subsequently underwent qualitative review by 10 nurses working in specialized wound-care clinics in the same autonomous community as the study setting. They assessed content relevance and question clarity, considered the content appropriate, and recommended changes limited to question wording.

For each image, evaluators selected one predefined response from seven domains: wound etiology, predominant wound-bed tissue, moisture or exudate level, visually apparent signs of infection, periwound condition, primary therapeutic objective, and primary dressing selection. The etiology question also allowed an “Other” response with free-text specification. The six etiology response categories available to nurses were pressure injury, venous leg ulcer, arterial or ischemic ulcer, diabetic foot ulcer, surgical wound (dehiscence or healing by secondary intention), and “Other,” which required a free-text description. Supplementary File S1 contains additional agreement and sensitivity analyses (Tables S1–S3), image-set characteristics (Tables S4 and S5), leave-one-nurse-out results (Table S6), and the technical configuration of the AI systems (Table S7). The questionnaire and archived Spanish model prompt, with an English translation, are provided in Supplementary File S2. The completed STROBE, GRRAS, and MI-CLEAR-LLM reporting checklists are provided in Supplementary File S3.

In June 2026, nurses accessed the questionnaire by scanning a QR code and independently evaluated the 30 wound images. We presented cases to all nurses in the same format and fixed sequence, without randomization or counterbalancing. Participants had no access to patient information, other nurses’ responses, or artificial intelligence outputs. One response was required for each domain.

### 2.6. Artificial Intelligence Systems and Evaluation

In June 2026, the researchers evaluated three general-purpose multimodal generative artificial intelligence models: GPT-5 (OpenAI), Gemini 2.5 Flash (Google), and Claude Sonnet 4.6 (Anthropic). All models were accessed through application programming interfaces (APIs) using a web application developed by the research team and operated from a desktop computer. The application was developed by the research team using Next.js and TypeScript specifically to support the study’s model evaluations. It served as an interface for submitting images and prompts to the model providers and retrieving their responses. The application is undergoing intellectual property registration through the University of León and has not been commercially sold or licensed. Potential research access to the application is subject to institutional authorization, agreement among the relevant rights holders, and written conditions governing its permitted use. No study-specific fine-tuning was performed.

In the study version, each model received an anonymized wound photograph and a standardized Spanish-language prompt covering the same seven assessment domains as the nurse questionnaire. During prompt development, we removed the “Other” etiology option from the model questionnaire to require selection from the five prespecified wound etiologies; the nurse questionnaire retained “Other” to allow free-text responses when none of those categories was considered appropriate. The final Spanish-language prompt used for the model evaluations is reproduced in Supplementary File S2. The application bundled the instructions and image into a single API request and requested structured output in JSON format. For each photograph, the researcher selected one model, obtained its response, reset the application, and repeated the procedure with another model. Each model evaluated each photograph once. The requests did not include prior assessments, nurses’ responses, or outputs generated by the other models. No follow-up prompts were issued, and the researcher did not revise or otherwise modify the model-generated responses.

The full archived Spanish prompt is reproduced in Supplementary File S2, Section S2.2; its English translation is provided in Section S2.3 solely for publication purposes and was not used during the experiment. All models received the Spanish-language instructions, with the seven assessment domains presented in the same fixed order: wound etiology, predominant wound-bed tissue, moisture or exudate level, visually apparent signs of infection, periwound condition, primary therapeutic objective, and primary dressing selection. The prompt required one predefined option per domain, using the exact wording supplied, without explanations, reasoning, or additional comments. The application returned the selected response options directly; researchers did not interpret free-text model outputs or manually assign them to response categories.

The instructions also assigned each model the role of a nurse specializing in acute and chronic wound care, with experience in pressure injuries, lower-extremity ulcers, diabetic foot ulcers, and surgical wounds. They explicitly referenced GNEAUPP and EWMA guidance and the TIME/TIMERS framework. They provided assessment rules on wound etiology, predominant tissue, moisture, visible signs of infection, and alignment between the therapeutic objective and dressing selection. A specific precaution addressed dry, stable necrosis in wounds of suspected arterial or ischemic origin. These were instructions supplied to the models and should not be interpreted as evidence that their responses complied with the referenced guidance. The complete Spanish instruction block and its English translation are included in Supplementary File S2.

We conducted model evaluations between 2 June and 16 June 2026. GPT-5 was accessed through OpenAI’s native Chat Completions API, Gemini 2.5 Flash through Google’s OpenAI-compatible endpoint, and Claude Sonnet 4.6 through Anthropic’s OpenAI SDK compatibility layer. Supplementary Table S7 reports the requested and returned model identifiers, provider-specific endpoints, generation settings, conversation context, execution dates, and runtime and client library versions. Raw API responses were retained, including the returned model identifiers and request timestamps.

### 2.7. Outcomes and Operational Definitions

For each case–domain task, inter-nurse agreement was summarized as the proportion of nurses selecting the most frequent response category (modal share); domain-level agreement was the mean modal share across the 30 cases. The main analyses retained modal shares as numerical values, reported with 95% confidence intervals, without categorization. For a secondary descriptive analysis, case–domain tasks were grouped according to their nurse modal share into three strata: ≥70%, 50% to <70%, and <50%. We specified these cutoffs before examining the study data as operational boundaries for describing the concentration of nurse responses. The 50% boundary distinguished tasks in which the most frequent response was selected by at least half of the panel from those in which it was selected by fewer than half; the 70% boundary further distinguished tasks with a larger concentration of responses in the modal category. These thresholds were not intended to define clinically validated levels of agreement, correctness, or acceptable performance. Although we prespecified the cutoffs, the stratified analysis remained secondary and exploratory.

For each wound image and questionnaire domain, we identified the response category nurses selected most often. When two or more categories tied for the highest frequency, all were retained in the nurse modal set. Each model’s response was compared with this set at the level of the response category within the corresponding domain. Concordance required the model-selected category to match any category in the nurse modal set; it did not require identical wording between the nurse questionnaire and the model prompt. The nurse modal set was an empirical comparator representing the most frequent nurse responses, not an independently established standard of clinical correctness. Throughout this report, inter-nurse agreement and intermodel agreement describe consistency within the respective evaluator groups, whereas concordance with the nurse modal set describes the model–comparator match. Complete intermodel agreement was defined as all three models selecting the same response category for a given wound image and assessment domain, regardless of whether that category belonged to the nurse modal set. Its overall proportion was calculated as the number of case–domain tasks meeting this criterion divided by the 210 evaluated tasks (30 images × seven domains). For the secondary descriptive stratified analysis, each case–domain task was classified by its human modal share. These comparisons established neither accuracy nor clinical validity.

To describe the image set’s visual composition, we summarized the nurses’ modal responses for predominant wound-bed tissue, moisture or exudate level, visually apparent signs compatible with infection, and periwound condition. For each domain, images were counted according to their modal category, with tied modal combinations reported separately to avoid double counting. We derived this descriptive characterization from the study ratings and did not use it as an independent clinical reference standard.

### 2.8. Statistical Analysis

Participant characteristics were summarized using frequencies and percentages or means and standard deviations. One author (A.G.-G.) reviewed the nurses’ free-text etiology responses before analysis. Nine responses that explicitly indicated a surgical origin were assigned to the prespecified surgical-wound category: “Herida quirúrgica sin dehiscencia” (*n* = 2), “Herida quirurgica” (*n* = 2), “Herida quirúrgica” (*n* = 2), “Incisión” (*n* = 2), and “Complicación intervencion quirurgica” (*n* = 1). This coding was based on surgical origin; the free-text responses did not necessarily establish dehiscence or healing by secondary intention. The remaining 25 responses were retained as “Other”; examples included burns (“quemadura”), cellulitis (“Celulitis”), laceration (“Laceracion”), and stump (“Muñón”). To assess the effect of this coding decision, we repeated the etiology agreement analysis with all 34 free-text responses retained as “Other,” without surgical recoding. We checked whether the nurse modal set changed for any image. The archived model prompt did not list “Other” as an etiology option, and none of the recorded model responses selected it.

The wound case was the sampling and resampling unit, whereas the case–domain combination defined model concordance. The same 18 nurses assessed all 30 wounds across seven domains, and each of the three models assessed the same images and domains. Thus, each model contributed 210 case–domain assessments, which were not treated as 210 independent wound cases. The nurse panel was retained as a fixed set of evaluators for the uncertainty analyses.

We summarized inter-nurse agreement using modal shares. Gwet’s AC1 was used as the primary chance-corrected agreement coefficient for nominal ratings because it is less sensitive than kappa to imbalanced category frequencies, which can produce low kappa values despite substantial observed agreement [23]. Fleiss’ kappa was also reported as a sensitivity measure. The coefficients use different definitions of chance-expected agreement; therefore, differences between their values reflect the chance-correction approach applied to the same ratings and should not be interpreted as contradictory observations or evidence of clinical accuracy. Both coefficients were interpreted alongside the modal shares.

Concordance with the nurse modal set and complete intermodel agreement were reported as proportions. Confidence intervals for human agreement statistics and overall concordance with the nurse modal set were estimated using 20,000 bootstrap samples drawn with replacement from the 30 wound cases. Each resampled case retained its nurse ratings and model responses across all seven domains. Resampling complete cases preserved within-wound dependence and the pairing of model assessments, rather than resampling individual ratings or case–domain observations independently. This approach incorporated within-wound dependence into the bootstrap uncertainty estimates without requiring an explicit parametric model of correlations between domains. The resulting intervals quantify variation across cases conditional on the participating nurse panel; they do not incorporate uncertainty from sampling a different panel of nurses. Domain-specific concordance proportions used Wilson 95% confidence intervals, with one concordance outcome per wound for each model and domain.

Overall model concordance was compared using paired differences, with the wound case as the unit of analysis. The seven domain assessments within each wound were retained together in the case-clustered bootstrap and case-level sign-permutation analyses; the 210 case–domain tasks were not treated as independent observations. Two-sided sign-permutation tests provided a nonparametric assessment of paired differences in overall concordance under the null assumption of exchangeability of the paired model outcomes within cases. We selected this approach to evaluate the observed difference in concordance without relying on a normal approximation for case-level differences or switching to a rank-based statistic. The *p*-values for the three overall pairwise model comparisons were adjusted using Holm’s method. Domain-specific results were descriptive and were reported as counts, proportions, and pointwise 95% confidence intervals, without domain-specific hypothesis tests comparing models. These intervals were not simultaneous confidence intervals and were not used to establish statistically significant differences between models within individual domains. All model comparisons were exploratory because they were based on only 30 purposively selected wound cases and were not intended to establish a generalizable ranking. Two sensitivity analyses examined the handling of tied nurse modal responses. The first retained all 210 case–domain tasks and selected the tied category with the lowest prespecified numerical code. The second excluded the 10 tasks with tied modal responses and calculated concordance across the remaining 200 tasks with a unique nurse modal response. The exclusion analysis was summarized descriptively using concordant counts and percentages. Analyses were performed using Python 3.12.13 and NumPy 2.3.5 with a fixed random seed. All analyses assessed agreement or concordance rather than clinical accuracy.

An exploratory leave-one-nurse-out analysis provided a descriptive human benchmark. For each case–domain task, we excluded one nurse and constructed the modal set from the responses of the remaining 17 nurses, retaining all categories tied for the highest frequency. The excluded nurse’s response was classified as concordant if it matched any category in this set. This procedure was repeated for all 18 nurses, using the same etiology recoding as in the main analysis. Each nurse’s concordance was calculated across 30 cases within each domain and across all 210 case–domain tasks. We summarized the distribution across nurses using the median, 25th and 75th percentiles, and minimum and maximum; we calculated percentiles using linear interpolation. Comparisons with model concordance were descriptive because the human benchmark used 17-nurse comparators, whereas model concordance used the full 18-nurse panel. No formal tests of human–model differences were performed.

### 2.9. Ethical Considerations

The study was conducted in accordance with the Declaration of Helsinki and applicable data-protection legislation. The Research Ethics Committee with Medicines of the Health Areas of León and El Bierzo (Comité de Ética de la Investigación con medicamentos de las Áreas de Salud de León y del Bierzo) approved the study on 28 April 2026 (reference 26044), before wound photographs were obtained in May 2026. Nurses and models completed assessments in June 2026. Nurses provided written informed consent before participation. Patients provided written informed consent for wound photography and the research use of their images, including submission of de-identified photographs to third-party commercial artificial intelligence providers. Participation was voluntary, and nurse responses were anonymous.

The protocol submitted for ethics review described and justified using commercial artificial intelligence services and included the patient information sheet, patient consent form, and nurse participation consent form; the approved protocol covered submitting de-identified wound photographs to these providers for study assessments.

We de-identified images and removed identifying metadata before evaluation. Study data were stored on a secure University of León server in accordance with Regulation (EU) 2016/679 and Spanish Organic Law 3/2018. Only de-identified wound images were submitted to the artificial intelligence systems; no patient names, personal identifiers, or accompanying clinical information were provided. Model outputs were generated exclusively for research and were not used to guide patient care.

## 3. Results

The image set comprised 30 wounds from 30 patients, with six cases (20.0%) in each of the five recruitment categories: pressure injuries, venous ulcers, arterial ulcers, diabetic foot ulcers, and surgical wounds healing by secondary intention (Table S4).

The visual characteristics attributed to the images by the nurse panel are summarized in Table S5. Necrotic tissue and slough were each the unique modal response for eight images (26.7%), followed by epithelialization tissue for seven (23.3%) and granulation tissue for four (13.3%). Fifteen images (50.0%) were predominantly rated as dry and nine (30.0%) as optimally moist. No visible signs of infection was the modal response for 11 images (36.7%), whereas suspected local infection and clear signs of local infection were the modal responses for six (20.0%) and five (16.7%), respectively. Maceration was the most frequent modal periwound category, occurring in 10 images (33.3%). Tied modal responses occurred in two images for tissue type and one image each for moisture and periwound condition.

Of the 30 eligible nurses invited, 18 participated (60%) and completed all 30 case assessments without missing data. Participants had a mean age of 42.8 years (SD 7.6); 15 were women (83.3%). Mean nursing experience was 18.5 years (SD 6.9), including 11.2 years (SD 5.4) of wound-care experience. Eleven participants worked primarily in hospital settings (61.1%), five in primary care (27.8%), and two in long-term or social health care (11.1%). All reported formal wound-care education: nine held a university expert qualification (50.0%), four reported a master’s degree in wound or skin care (22.2%), and five had completed accredited continuing education (27.8%) (Table 1). Experience in specific wound-care areas or individual wound etiologies was not collected.

Values are n (%) unless otherwise indicated. Academic-degree categories were not mutually exclusive. SD, standard deviation.

After characterizing the nurse sample, we examined agreement in wound assessment and treatment-related decisions across the seven prespecified domains (Table 2).

Wound etiology showed the highest agreement, with a mean modal share of 71.3% (95% CI 63.3–79.4) and a Gwet’s AC1 of 0.520 (95% CI 0.398–0.651). Agreement was lower for predominant tissue and exudate/moisture, although both had a mean modal share of 65.6%. Periwound condition, signs compatible with infection, and the primary therapeutic objective showed greater variability. Primary dressing selection showed the lowest modal share (43.7%, 95% CI 39.6–48.0) and the lowest Fleiss’ kappa (0.095, 95% CI 0.061–0.121). The broadly consistent pattern across the three agreement measures indicated that variability was greatest in clinically interpretive and treatment-related decisions.

In the etiology coding sensitivity analysis, retaining all 34 free-text responses as “Other” reduced the mean nurse modal share from 71.3% to 69.6% and Gwet’s AC1 from 0.520 to 0.483. The nurse modal set remained unchanged for all 30 images. Consequently, concordance with the nurse modal set for the etiology domain—and the reported overall model concordance—was unchanged under this alternative coding. This sensitivity analysis addresses recoding nurse responses but does not remove differences between the response options documented for nurses and models.

Given the variability in nurse judgments, we compared each artificial intelligence model with the nurse modal set across the same seven assessment domains (Table 3).

Across the 30 wound cases and seven assessment domains evaluated in this experiment, overall concordance with the nurse modal set was 63.3% for Gemini (133/210; 95% CI 56.7–70.0), 48.6% for ChatGPT (102/210; 95% CI 40.5–56.2), and 44.8% for Claude (94/210; 95% CI 38.6–51.4). Domain-specific estimates showed variation that was not captured by these overall proportions (Table 3). Concordance ranged from 23.3% to 46.7% across models for signs compatible with infection, from 40.0% to 43.3% for periwound condition, from 46.7% to 66.7% for the primary therapeutic objective, and from 30.0% to 46.7% for primary dressing selection. Wound etiology concordance ranged from 60.0% to 86.7%. These estimates describe the responses obtained under the evaluated image-only conditions.

In the exploratory leave-one-nurse-out analysis, overall nurse concordance had a median of 57.1% (25th–75th percentiles, 53.6–61.4%) and ranged from 49.0% to 63.3% across the 18 nurses (Table S6). Domain-specific median concordance ranged from 36.7% for primary dressing selection to 68.3% for wound etiology. For descriptive context, Gemini’s overall concordance with the full nurse modal set was 63.3%, matching the highest observed individual nurse concordance, whereas ChatGPT’s 48.6% and Claude’s 44.8% fell below the observed nurse range. These comparisons used different comparator panel sizes and do not establish clinical equivalence or superiority.

To examine whether model–nurse alignment varied with the consistency of human judgment, concordance was stratified by task-level inter-nurse agreement (Table 4).

Among tasks with a nurse modal share ≥70%, concordance with the nurse modal set was 91.5% for Gemini, 78.7% for ChatGPT, and 63.8% for Claude. Corresponding values were 64.6%, 41.4%, and 35.4% in the 50% to <70% stratum, and 40.6%, 37.5%, and 45.3% in the <50% stratum. Concordance decreased across these strata for ChatGPT and Gemini, whereas Claude showed no monotonic pattern. These secondary descriptive comparisons characterize concordance across numerical strata of nurse response concentration; they do not identify levels of clinical quality. We derived the strata and comparator from the same nurse responses, which limits interpretation of their association.

To further characterize agreement patterns and assess the robustness of the main findings, we examined intermodel agreement, paired model comparisons, and the handling of tied nurse responses in supplementary analyses. Complete agreement among the three artificial intelligence models was observed in 81 of 210 case–domain tasks (38.6%; Table S1). It was highest for wound etiology (18/30, 60.0%) and lowest for signs compatible with infection (5/30, 16.7%).

Exploratory paired comparisons showed that Gemini had 14.8 percentage points greater concordance with the nurse modal set than ChatGPT (95% CI 5.7–24.3; Holm-adjusted *p* = 0.011) and 18.6 percentage points greater concordance than Claude (95% CI 10.5–26.7; Holm-adjusted *p* < 0.001; Table S2). The difference between ChatGPT and Claude was smaller and not statistically significant (3.8 percentage points, 95% CI −3.8 to 11.0; *p* = 0.266).

Ten of the 210 case–domain tasks (4.8%) contained two nurse response categories tied for the highest frequency: one in wound etiology, two in predominant wound-bed tissue, one in exudate/moisture, none in signs compatible with infection, one in periwound condition, two in the primary therapeutic objective, and three in primary dressing selection.

The deterministic tie-breaking analysis retained all 210 tasks and yielded concordance estimates of 47.6% for ChatGPT, 61.4% for Gemini, and 43.3% for Claude (Table S3). Among the 10 tied tasks, ChatGPT, Gemini, and Claude matched a category in the nurse modal set in seven, six, and eight tasks, respectively. Excluding these tasks yielded concordance estimates of 95/200 (47.5%) for ChatGPT, 127/200 (63.5%) for Gemini, and 86/200 (43.0%) for Claude. Relative to the primary analysis, these represented changes of −1.1, +0.2, and −1.8 percentage points, respectively. Both sensitivity analyses preserved the observed ordering of overall model concordance. These findings indicate that tasks with tied modal responses did not drive the descriptive pattern; they do not establish clinical correctness or the statistical significance of between-model differences after exclusion.

## 4. Discussion

This study examined variability in image-based wound assessment and treatment-related decisions among nurses with wound-care expertise and quantified each model’s concordance with the nurse modal set. Inter-nurse agreement varied across domains, with the greatest dispersion in responses for primary dressing selection. Model concordance also varied across domains and evaluated systems within this dataset. These findings describe response consistency and concordance with the most frequent nurse responses under the study conditions. They do not establish which responses were clinically correct or whether any model was clinically superior.

When interpreting these findings, we must distinguish agreement, concordance, accuracy, and clinical validity. Agreement and concordance describe relationships among responses; accuracy concerns whether a response is correct against an independent reference standard, while clinical validity concerns whether an assessment or recommendation is appropriate for its intended clinical use. Nurses and models assessed the same images across the same seven domains, enabling comparison of their selected response categories. However, assessment conditions were not fully identical: the nurse questionnaire included an “Other” etiology option that was unavailable to the models, and the instruments differed in wording and accompanying instructions. These differences limit comparability, and the nurse modal set does not establish clinical correctness. A concordant model response could therefore reflect either a correct assessment or a shared error. Conversely, a nonconcordant response could represent either a model error or a clinically justified alternative. Without independent adjudication informed by the relevant clinical context, these possibilities cannot be distinguished. Therefore, higher concordance should not be interpreted as greater accuracy, clinical validity, or suitability for patient care.

The pattern of inter-nurse agreement is consistent with evidence that visual wound assessment is not fully reproducible. Ramachandram et al. reported variability in clinicians’ identification and estimation of wound-bed tissue [24], while Vains and Finlayson described multiple sources informing generalist nurses’ wound-management decisions [25]. In the present study, missing information on pain, odor, perfusion, treatment history, and previous response may have widened the range of clinically defensible choices [2]. Research on artificial intelligence in home wound care also emphasizes the importance of clinical workflows and professional judgment [26]. The variability in dressing selection may therefore reflect both ambiguity in the photographs and differences in clinical practice, including the experience and decision-making approaches individual nurses bring to the task. These explanations are not mutually exclusive, and the study was not designed to determine their relative contributions.

The nurse modal set identifies the most frequently selected response or responses within this panel under the study conditions. It therefore represents a typical choice within the evaluated task, rather than an established optimal choice for the patient. Determining the most appropriate dressing would require consideration of the wound and patient context, treatment goals, and relevant clinical evidence [2]. A frequent response may reflect a shared interpretation or practice preference rather than the best option for an individual patient; conversely, a less frequent response may be clinically defensible. Human disagreement should therefore be distinguished from model error: dispersion among nurse responses reflects variability within the panel, while model nonconcordance reflects a deviation from the panel’s modal set. Neither observation alone identifies an incorrect assessment. Without an independent reference standard informed by clinical context, the study cannot distinguish model error from a clinically justified alternative or determine whether a concordant response reflects a shared error.

The leave-one-nurse-out analysis provides a descriptive reference for interpreting model concordance alongside variability among individual nurses. Excluding each nurse from their own comparator avoids incorporating that nurse’s response into the modal set against which it is evaluated. However, the human concordance estimates remain dependent because the comparator panels overlap, and the human and model comparisons use panels of different sizes. Moreover, binary modal-set concordance does not distinguish a narrow majority from unanimous agreement. The additional analysis therefore contextualizes concordance without resolving the absence of a clinical reference standard or replacing assessment of the underlying response distributions.

Variation in individual nurse concordance aligns with Mohammed et al. [5], who reported differences in inter-clinician agreement across wound tissue types, particularly for epithelialization. More directly relevant to treatment decisions, Jørgensen et al. [27] found lower agreement on treatment recommendations, including dressing selection, than on ulcer classification when clinicians assessed 30 diabetic foot ulcer photographs. Our finding that dressing selection had the lowest median leave-one-nurse-out concordance adds evidence of variability in image-based therapeutic choices. Together with the multiple influences on wound-management decisions identified by Vains and Finlayson [25], these findings support contextualizing model concordance against human variability. However, differences in assessment tasks and statistical measures preclude direct numerical comparison.

The domain-specific findings warrant particular attention because the overall concordance proportion combines assessment tasks with different clinical purposes and information requirements. Mean nurse modal shares were 52.4% for signs compatible with infection, 59.3% for periwound condition, 54.8% for the primary therapeutic objective, and 43.7% for primary dressing selection. These findings describe variability among nurses, whereas the corresponding model concordance estimates describe agreement with the nurse modal set; neither establishes clinical correctness. In particular, the infection domain concerned visually apparent findings rather than a confirmed diagnosis. Image-only assessment was not considered sufficient to establish the appropriateness of patient-specific treatment decisions. The photographs were presented without the clinical history, perfusion findings, symptoms, or previous treatment response needed for a fully informed therapeutic assessment [2]. Concordance in the therapeutic-objective and dressing-selection domains therefore indicates agreement with the panel’s most frequently selected response categories under these restricted conditions, rather than evidence that the selected treatment was appropriate for the patient. Low concordance may reflect ambiguity arising from incomplete information or differences in clinical judgment, rather than necessarily indicating model error. However, the study cannot determine the relative contributions of task ambiguity, variation in professional judgment, and model error, nor establish that any particular nonconcordant response was clinically justified. The recorded choices should therefore be interpreted as responses to a constrained visual task, not validated treatment recommendations.

The present findings extend the evidence on variability in image-based wound assessment by examining how model responses align with a nurse-derived comparator. Jørgensen et al. [27] found lower interobserver agreement for treatment recommendations, including dressing selection, than for ulcer classification, consistent with the variability observed in our study. Such variability helps contextualize model–nurse concordance but does not establish whether either assessment is clinically correct. By comparison, Apichonbancha et al. [13] evaluated 450 wound photographs against an expert consensus reference standard established by three clinicians. Their evaluation addressed agreement with an explicitly established reference, whereas our study measured alignment with the most frequently selected responses of a nurse panel without independent adjudication. These methodological differences prevent us from interpreting their accuracy estimates and our concordance estimates as equivalent measures. Differences in model versions, image sets, prompts, response constraints, and reference standards further limit comparisons across studies [28,29]. Specifically, Gemini’s concordance of 63.3% represents 133 matches with the nurse modal set across 210 case–domain tasks; it does not indicate that 63.3% of clinical decisions were correct. Although the leave-one-nurse-out analysis provides a descriptive human benchmark, neither the observed nurse range nor the studies discussed here establishes a clinically acceptable threshold for our concordance measure. For clinicians and policymakers, these findings describe response alignment under the evaluated conditions. Establishing clinical acceptability would require evaluation against an appropriate independent reference standard, with criteria reflecting the intended clinical use and the consequences of different errors.

The stratified analysis in Table 4 is retained solely as a secondary description of the observed responses. Both stratum assignment and the modal comparator were derived from the same nurse ratings, so the analysis does not provide an independent test of whether models perform better on clinically unambiguous cases. Nurse modal share was not an externally validated measure of case difficulty, and the observed association should not be interpreted as evidence of diagnostic accuracy or an ability to resolve clinical uncertainty. Uncertainty in human annotations can affect the evaluation of clinical artificial intelligence [30]. Future studies could separate agreement-strata construction and comparator selection using independent panels or assess case difficulty with prespecified external criteria; evaluating clinical correctness would also require an appropriate independent reference standard.

The supplementary analyses further characterized the responses obtained in this experiment. Complete intermodel agreement occurred in 38.6% of case–domain tasks, indicating that the three models frequently selected different responses under the evaluated conditions. The exploratory paired comparisons quantified differences in concordance within this dataset, while alternative handling of tied nurse responses had little influence on the overall estimates. These findings do not establish a stable hierarchy of models: the observed differences depend on the selected images, participating nurse panel, model versions, prompt, and a single evaluation of each image by each model. Their persistence under other evaluation conditions remains to be established.

### 4.1. Limitations

Only 18 of the 30 invited nurses completed the assessment, and we did not investigate the reasons for nonparticipation among the remaining 12. Because sociodemographic and professional characteristics were unavailable for the 12 nonparticipants, we could not compare participants and nonparticipants. Nonresponse bias cannot be excluded: nurses who completed the assessment may have differed from nonparticipants in ways relevant to their wound assessments. The direction and magnitude of any such bias remain unknown. Similarly, the 100% patient consent rate indicates acceptance among those approached but does not establish representativeness of the purposively selected wound sample. Shared regional practice patterns and professional backgrounds may have favored agreement on some response categories, although the direction and magnitude of this influence cannot be determined from our data. Agreement estimates may differ in panels with other training backgrounds and practice settings, while differences in case mix and photography conditions may also affect model–nurse concordance. Equal allocation provided coverage across wound categories but did not reflect their prevalence or full clinical and visual spectrum. Selection based on wound category, visual presentation, and anonymity considerations may have introduced selection bias. The small number of cases per category limits subgroup interpretation, and differences in case composition could alter the overall concordance estimates. The bootstrap analyses retained the same nurse panel in every resample; therefore, their confidence intervals reflect variation across wound cases conditional on these evaluators and do not quantify how the findings might vary with a different panel of nurses.

The image-only design omitted important clinical information, and the nurse modal set was not an independently adjudicated reference standard. Consequently, the study could not distinguish correct concordance with the nurse modal set from shared error or determine whether nonconcordant model responses were clinically justified. This limits interpretation of all model comparisons to concordance with the nurse modal set, without establishing accuracy or clinical validity. Each model assessed each image only once; therefore, within-model response stability and test–retest reliability could not be evaluated. Repeated requests using the same image and prompt may produce different responses from a generative model. Consequently, the reported concordance estimates characterize the responses obtained in the study rather than demonstrating stable model behavior across repeated assessments. The observed differences between models may also be sensitive to response variability. The case-clustered confidence intervals quantify uncertainty across the sampled cases but do not capture variability arising from repeated model outputs for the same case. Future studies should include repeated, independent assessments of the same images under standardized conditions, document model versions and generation settings, and quantify within-model agreement for each assessment domain. This would help determine whether both individual responses and comparative concordance patterns are reproducible.

Section 2.6 reports the model identification, access method, and evaluation period. The findings remain specific to the evaluated systems and experimental conditions and should not be assumed to generalize to subsequent model releases. The nurse questionnaire offered an “Other” etiology response, whereas the archived model prompt did not list this option. This asymmetry may have constrained model responses and limits direct interpretation of etiology concordance. In addition, one author recoded nurse free-text responses into the surgical-wound category without independent adjudication, although retaining all free-text responses as “Other” did not change any case-level nurse modal set. This sensitivity analysis addresses only the recoding of nurse responses; it neither eliminates the asymmetry in available response options nor establishes how the models would have responded had “Other” been available. Unchanged concordance under alternative nurse-response coding therefore does not demonstrate equivalent evaluation conditions. Etiology-specific concordance and the overall concordance estimates that include this domain must be interpreted in light of this unresolved limitation. Future evaluations should provide equivalent response options to human and model evaluators. We derived the agreement strata and comparator from the same responses, and the subgroup and pairwise comparisons remain exploratory.

All nurses assessed the 30 cases in the same fixed sequence. Consequently, order effects, including familiarization with the task or fatigue during successive assessments, cannot be excluded. We did not evaluate these effects, and their potential influence on nurse responses and the resulting modal comparator remains unknown.

### 4.2. Future Research

Future research should evaluate these models in larger, multicenter datasets encompassing diverse wound types, patient populations, and clinical settings, using independently adjudicated reference standards. Studies should also examine whether incorporating relevant clinical information improves performance and reduces variability across models, prompts, and repeated assessments. Finally, prospective evaluations are needed to determine their effects on nurses’ decision-making, patient safety, workflow, and clinical outcomes before implementation in routine wound care.

## 5. Conclusions

Image-based wound assessment and treatment decisions varied meaningfully even among expert nurses. Agreement was highest for wound etiology, intermediate for tissue and moisture assessment, and weaker for infection signs and periwound condition; variability was particularly evident in defining therapeutic objectives and selecting a primary dressing. Concordance with the nurse modal set varied across models and assessment domains within the evaluated dataset. Gemini showed the highest overall concordance with the nurse modal set in this dataset, but this finding does not establish greater accuracy or clinical validity. Concordance with the nurse modal set reflects agreement with the panel’s most frequently selected responses rather than evidence of clinical correctness. Without an independent clinical reference standard, concordant responses may represent shared errors, whereas nonconcordant responses may represent clinically appropriate alternatives. These findings characterize response patterns under image-only conditions and support further investigation using clinical context and independent adjudication before drawing conclusions about the models’ suitability for wound-care decisions.

Further studies assessing clinical accuracy, safety, and patient outcomes are needed to determine whether these models can support wound assessment and treatment selection.

## Figures and Tables

**Table 1:** Participant characteristics.

Characteristic	Category or Statistic	Value
Sex	Women	15 (83.3%)
	Men	3 (16.7%)
Age, years	Mean (SD)	42.8 (7.6)
Nursing experience, years	Mean (SD)	18.5 (6.9)
Wound-care experience, years	Mean (SD)	11.2 (5.4)
Primary clinical setting	Hospital	11 (61.1%)
	Primary care	5 (27.8%)
	Long-term or social health care	2 (11.1%)
Wound-care education	University expert program	9 (50.0%)
	Master’s-level wound or skin-integrity study	4 (22.2%)
	Accredited continuing education	5 (27.8%)
Academic degree	Bachelor’s degree	18 (100.0%)
	Master’s degree	8 (44.4%)
	Doctoral degree	0 (0.0%)

**Table 2:** Agreement among experienced nurses by assessment domain.

Assessment Domain	Mean Modal Share, % (95% CI)	Gwet’s AC1 (95% CI)	Fleiss’ κ (95% CI)
Wound etiology	71.3 (63.3–79.4)	0.520 (0.398–0.651)	0.475 (0.351–0.586)
Predominant wound-bed tissue	65.6 (58.7–72.8)	0.395 (0.297–0.507)	0.386 (0.283–0.475)
Exudate/moisture level	65.6 (58.5–72.8)	0.388 (0.274–0.512)	0.321 (0.223–0.405)
Signs compatible with infection	52.4 (47.8–57.6)	0.165 (0.104–0.245)	0.149 (0.091–0.202)
Periwound condition	59.3 (54.1–64.6)	0.272 (0.205–0.352)	0.243 (0.166–0.314)
Primary therapeutic objective	54.8 (50.7–59.3)	0.202 (0.156–0.262)	0.197 (0.141–0.249)
Primary dressing selection	43.7 (39.6–48.0)	0.163 (0.121–0.208)	0.095 (0.061–0.121)

The modal share is the proportion selecting the most frequent response category for each case, averaged across 30 cases. AC1, agreement coefficient 1; CI, confidence interval; κ, kappa. Agreement-statistic intervals were estimated by case bootstrap.

**Table 3:** Concordance of each artificial intelligence model with the nurse modal set.

Assessment Domain	ChatGPT, n/N; % (95% CI)	Gemini, n/N; % (95% CI)	Claude, n/N; % (95% CI)
Wound etiology	18/30; 60.0 (42.3–75.4)	26/30; 86.7 (70.3–94.7)	21/30; 70.0 (52.1–83.3)
Predominant wound-bed tissue	15/30; 50.0 (33.2–66.8)	21/30; 70.0 (52.1–83.3)	14/30; 46.7 (30.2–63.9)
Exudate/moisture level	19/30; 63.3 (45.5–78.1)	25/30; 83.3 (66.4–92.7)	12/30; 40.0 (24.6–57.7)
Signs compatible with infection	11/30; 36.7 (21.9–54.5)	14/30; 46.7 (30.2–63.9)	7/30; 23.3 (11.8–40.9)
Periwound condition	13/30; 43.3 (27.4–60.8)	13/30; 43.3 (27.4–60.8)	12/30; 40.0 (24.6–57.7)
Primary therapeutic objective	17/30; 56.7 (39.2–72.6)	20/30; 66.7 (48.8–80.8)	14/30; 46.7 (30.2–63.9)
Primary dressing selection	9/30; 30.0 (16.7–47.9)	14/30; 46.7 (30.2–63.9)	14/30; 46.7 (30.2–63.9)
All domains	102/210; 48.6 (40.5–56.2)	133/210; 63.3 (56.7–70.0)	94/210; 44.8 (38.6–51.4)

Concordance required an exact match with any response in the nurse modal set, including tied modes. Domain-specific intervals are Wilson 95% CIs; overall intervals use case-clustered bootstrap resampling. CI, confidence interval; n/N, concordant responses/evaluated tasks.

**Table 4:** Exploratory concordance with the nurse modal set by nurse modal share stratum.

Nurse Modal Share Stratum	Tasks, N	ChatGPT, n/N; % (95% CI)	Gemini, n/N; % (95% CI)	Claude, n/N; % (95% CI)
≥70%	47	37/47; 78.7 (63.4–91.2)	43/47; 91.5 (81.0–100.0)	30/47; 63.8 (45.0–80.6)
50% to <70%	99	41/99; 41.4 (29.5–54.3)	64/99; 64.6 (54.8–74.2)	35/99; 35.4 (27.2–44.4)
<50%	64	24/64; 37.5 (27.1–48.4)	26/64; 40.6 (31.4–50.0)	29/64; 45.3 (36.6–55.0)

Strata were defined using the nurse modal share for each case–domain task and were used solely for secondary descriptive analysis. The numerical boundaries do not represent validated levels of clinical quality, correctness, or acceptable agreement. Domain-level mean modal shares and their 95% confidence intervals are presented without categorization in Table 2. CI, confidence interval; n/N, concordant responses/tasks within each stratum. The strata and modal comparator were derived from the same nurse responses; therefore, the observed association should not be interpreted as independent validation of model responses.

## Data Availability

The study materials are undergoing intellectual property registration through the University of León and are not currently deposited in a public repository. Requests for access to de-identified nurse ratings, model responses, the category codebook, and analysis code should be addressed to the corresponding author and include a reasoned description of the proposed research or verification purpose and the materials requested. Requests will be reviewed by the University of León in consultation with the relevant rights holders, taking into account participant confidentiality, consent provisions, and intellectual property rights. Access is subject to institutional authorization and, where applicable, an agreement specifying permitted uses and conditions for handling and sharing the materials. Clinical wound photographs are considered separately and are not publicly available because of participant privacy and consent restrictions.

## Supplementary Materials

The following supporting information can be downloaded at https://www.mdpi.com/article/10.3390/healthcare14213587/s1, Supplementary File S1 contains Table S1 (complete intermodel agreement), Table S2 (pairwise differences in concordance with the nurse modal set), Table S3 (sensitivity analysis for tied nurse modal responses), Table S4 (wound-category composition of the purposively selected image set), Table S5 (image characteristics based on nurses’ modal responses), Table S6 (leave-one-nurse-out concordance and descriptive comparison with model concordance), and Table S7 (technical configuration of the AI systems evaluated in the study). Supplementary File S2 contains the complete nurse questionnaire in English, the Spanish-language model prompt and its English translation, all response categories, and the coding rules and procedures used to harmonize free-text etiology responses. Supplementary File S3 contains the completed STROBE, GRRAS, and MI-CLEAR-LLM reporting checklists.

## Abbreviations

AC1, agreement coefficient 1; AI, artificial intelligence; CI, confidence interval; GRRAS, Guidelines for Reporting Reliability and Agreement Studies; LLM, large language model; MI-CLEAR-LLM, Minimum Reporting Items for Clear Evaluation of Accuracy Reports of Large Language Models in Healthcare; PWA, progressive web application; SD, standard deviation; STROBE, Strengthening the Reporting of Observational Studies in Epidemiology.
