# Supplementary material for: Variability in Nurses’ Assessments and Concordance of Multimodal Generative AI Models in Image-Based Wound Assessment
Source: Healthcare (Basel). 2026 Oct 23;14(21):3587. doi: 10.3390/healthcare14213587 (PMC13763401; doi:10.3390/healthcare14213587)
Supplement: Supplementary file 1 [file healthcare-14-03587-s001.zip › Supplementary_File_S1_Tables.pdf]

Supplementary File S1

Variability in nurses′ assessments and concordance of multimodal generative AI models in image-based wound assessment

Additional agreement analyses

Table S1. Complete agreement among the three artificial intelligence models

Assessment domain	All three models identical, n/N; % (95% CI)	Gwet’s AC1 (95% CI)
Wound etiology	18/30; 60.0 (42.3–75.4)	0.649 (0.481–0.805)
Predominant wound-bed tissue	11/30; 36.7 (21.9–54.5)	0.420 (0.257–0.589)
Exudate/moisture level	9/30; 30.0 (16.7–47.9)	0.344 (0.191–0.513)
Signs compatible with infection	5/30; 16.7 (7.3–33.6)	0.254 (0.146–0.388)
Periwound condition	14/30; 46.7 (30.2–63.9)	0.558 (0.387–0.717)
Primary therapeutic objective	15/30; 50.0 (33.2–66.8)	0.523 (0.347–0.701)
Primary dressing selection	9/30; 30.0 (16.7–47.9)	0.428 (0.291–0.571)
All domains	81/210; 38.6%	—

Complete agreement required ChatGPT, Gemini, and Claude to select the same response category, irrespective of the nurse modal set. Wilson 95% confidence intervals accompany domain-specific proportions. Gwet’s AC1 estimates include case-bootstrap 95% confidence intervals. A pooled AC1 was not calculated because response categories differed across domains. AC1, agreement coefficient 1; CI, confidence interval; n/N, number of agreements/number of evaluated tasks.

Table S2. Pairwise differences in concordance with the nurse model set

Model comparison	Difference, percentage points (95% CI)	Unadjusted p value	Holm-adjusted p value
Gemini − ChatGPT	14.8 (5.7–24.3)	0.006	0.012
Gemini − Claude	18.6 (10.5–26.7)	0.0000057	0.0000171
ChatGPT − Claude	3.8 (−3.8 to 11.0)	0.266	0.266

Positive differences indicate higher concordance for the first model in each comparison. Confidence intervals were obtained using bootstrap resampling clustered by wound case. Unadjusted p-values were obtained from exploratory two-sided case-level sign-permutation tests. The Holm procedure was applied across the three pairwise comparisons. These comparisons assess concordance with the nurse modal set and do not establish clinical superiority. CI, confidence interval.

Table S3. Sensitivity analysis for tied nurse modal responses

Model	Tie-inclusive modal set, n/N; % (95% CI)	Deterministic tie-breaking rule, n/N; % (95% CI)	Reduction after tie-breaking, percentage points	Unique-mode tasks only, n/N; %
ChatGPT	102/210; 48.6 (40.5–56.2)	100/210; 47.6 (39.5–55.2)	1.0	95/200; 47.5
Gemini	133/210; 63.3 (56.7–70.0)	129/210; 61.4 (54.8–68.1)	1.9	127/200; 63.5
Claude	94/210; 44.8 (38.6–51.4)	91/210; 43.3 (36.7–50.0)	1.4	86/200; 43.0

Ten of the 210 case–domain tasks (4.8%) had two categories tied for the highest nurse response frequency: wound etiology, n = 1; predominant wound-bed tissue, n = 2; exudate/moisture, n = 1; signs compatible with infection, n = 0; periwound condition, n = 1; primary therapeutic objective, n = 2; and primary dressing selection, n = 3. The primary analysis retained both tied categories in the nurse modal set. The deterministic tie-breaking analysis selected the tied category with the lowest prespecified numerical code and retained all 210 tasks. We calculated reductions by subtracting the tie-breaking estimate from the primary estimate, using unrounded proportions and rounding to one decimal place. The unique-mode analysis excluded all 10 tied tasks, leaving 200 tasks per model. ChatGPT, Gemini, and Claude had seven, six, and eight concordant responses, respectively, among the excluded tasks. Confidence intervals for the primary and deterministic tie-breaking analyses were obtained using bootstrap resampling clustered by wound case. The unique-mode analysis is reported descriptively as counts and percentages, without confidence intervals or hypothesis tests. Concordance does not establish clinical correctness. CI, confidence interval; n/N, concordant responses/

Table S4. Wound-category composition of the purposively selected image set

Wound category used for recruitment	Cases, n	Percentage (%)
Pressure injuries	6	20.0
Venous ulcers	6	20.0
Arterial ulcers	6	20.0
Diabetic foot ulcers	6	20.0
Surgical wounds healing by secondary intention	6	20.0
**Total**	**30**	**100.0**

Each case represented one wound from one patient. Categories reflect the recruitment quotas reported by the investigators, not diagnoses established through independent adjudication or classifications derived from the nurse modal set. Equal allocation was intended to provide coverage across wound categories and does not reflect their prevalence in clinical practice.

Table S5. Image characteristics based on nurses' modal responses

Assessment domain	Modal response category	Images, n (%)
Predominant wound-bed tissue	Necrotic tissue	8 (26.7)
Predominant wound-bed tissue	Slough	8 (26.7)
Predominant wound-bed tissue	Epithelialization tissue	7 (23.3)
Predominant wound-bed tissue	Granulation tissue	4 (13.3)
Predominant wound-bed tissue	Mixed tissue without clear predominance	1 (3.3)
Predominant wound-bed tissue	Tied modes: epithelialization and granulation	1 (3.3)
Predominant wound-bed tissue	Tied modes: granulation and slough	1 (3.3)
Moisture/exudate level	Dry	15 (50.0)
Moisture/exudate level	Optimally moist	9 (30.0)
Moisture/exudate level	Macerating	3 (10.0)
Moisture/exudate level	Wet/saturated	2 (6.7)
Moisture/exudate level	Tied modes: wet/saturated and macerating	1 (3.3)
Visually apparent signs compatible with infection	No visible signs of infection	11 (36.7)
Visually apparent signs compatible with infection	Mild inflammation/local erythema	8 (26.7)
Visually apparent signs compatible with infection	Suspected local infection	6 (20.0)
Visually apparent signs compatible with infection	Clear signs of local infection	5 (16.7)
Periwound condition	Macerated	10 (33.3)
Periwound condition	Healthy/intact	8 (26.7)

Assessment domain	Modal response category	Images, n (%)
Periwound condition	Erythematous/inflamed	8 (26.7)
Periwound condition	Hyperkeratotic/callused	3 (10.0)
Periwound condition	Tied modes: healthy/intact and erythematous/inflamed	1 (3.3)

Categories summarize the modal responses of 18 nurses assessing each of the 30 images. Each image contributes once within each domain. When categories tied for the highest response frequency, the tied combination is reported separately rather than assigning a single category. Percentages use 30 images as the denominator and may not sum to 100% because of rounding. These classifications describe the panel's image-based assessments; they are not independently adjudicated clinical findings. In particular, infection-related categories reflect perceived visual signs rather than confirmed infection.

Table S6. Leave-one-nurse-out concordance and descriptive comparison with model concordance

Assessment domain	Nurse concordance, median % (P25–P75)	Nurse concordance, minimum–maximum %	ChatGPT, %	Gemini, %	Claude, %
Wound etiology	68.3 (66.7–75.8)	60.0–83.3	60.0	86.7	70.0
Predominant wound-bed tissue	66.7 (54.2–70.0)	40.0–76.7	50.0	70.0	46.7
Exudate/moisture level	66.7 (57.5–72.5)	50.0–76.7	63.3	83.3	40.0
Signs compatible with infection	55.0 (43.3–59.2)	36.7–70.0	36.7	46.7	23.3
Periwound condition	58.3 (53.3–63.3)	40.0–73.3	43.3	43.3	40.0
Primary therapeutic objective	51.7 (45.0–60.0)	26.7–73.3	56.7	66.7	46.7
Primary dressing selection	36.7 (30.8–50.0)	23.3–63.3	30.0	46.7	46.7
All domains	57.1 (53.6–61.4)	49.0–63.3	48.6	63.3	44.8

For each of the 18 nurses, concordance was calculated against the modal set of the other 17 nurses, retaining all tied modal categories. Domain-specific estimates used 30 tasks per nurse; overall estimates used 210 tasks per nurse. The main-analysis recoding of nine free-text etiology responses as surgical wounds was retained. P25 and P75 are the 25th and 75th percentiles across nurses, calculated using linear interpolation; they describe the observed distribution and are not confidence limits. Model estimates are reproduced from Table 3 and use the full 18-nurse model set. Human–model comparisons are descriptive because comparator panel sizes differ and the nurse estimates involve overlapping panels. Concordance does not establish clinical accuracy.

Supplementary Table S7. Technical configuration of the AI systems evaluated in the study.

Parameter	GPT-5	Gemini 2.5 Flash	Claude Sonnet 4.6
**Provider**	OpenAI	Google	Anthropic
**API model identifier requested**	gpt-5	gemini-2.5-flash	claude-sonnet-4-6
**Model identifier returned in stored responses**	gpt-5-2025-08-07	gemini-2.5-flash	claude-sonnet-4-6
**Endpoint (POST)**	https://api.openai.com/v1/chat/completions	https://generativelanguage.googleapis.com/v1beta/openai/chat/completions	https://api.anthropic.com/v1/chat/completions
**Interface**	Native Chat Completions API	OpenAI-compatible Gemini API endpoint	OpenAI SDK compatibility layer of the Claude API
**Temperature**	1.0	1.0; provider default, not modified	1.0; provider default, not modified
**Top-p**	Provider default	Not explicitly set	Not explicitly set
**Maximum output tokens**	Not explicitly set	Not explicitly set	Not explicitly set
**Reasoning / thinking**	reasoning_effort: medium (provider default)	Dynamic thinking (provider default)	Extended thinking not enabled
**Runs per case**	1	1	1

**Conversation context**	Independent request for each case; no prior conversation history, memory, or retrieval tools	Independent request for each case; no prior conversation history, memory, or retrieval tools	Independent request for each case; no prior conversation history, memory, or retrieval tools
**Seed**	Not explicitly set	Not explicitly set	Not explicitly set
**System prompt and input**	Identical assessment instructions and case input across models; full prompt in Supplementary Material S2	Identical assessment instructions and case input across models; full prompt in Supplementary Material S2	Identical assessment instructions and case input across models; full prompt in Supplementary Material S2
**Output logging**	Raw API responses stored, including returned model identifiers and request timestamps	Raw API responses stored, including returned model identifiers and request timestamps	Raw API responses stored, including returned model identifiers and request timestamps
**Execution dates**	June 2–16, 2026	June 2–16, 2026	June 2–16, 2026
**Application interface**	Investigator-developed web application built with Next.js and TypeScript	Investigator-developed web application built with Next.js and TypeScript	Investigator-developed web application built with Next.js and TypeScript
**API request execution environment and client library**	Python 3.12 component using the OpenAI Python SDK (openai), version 2.40.0	Python 3.12 component using the OpenAI Python SDK (openai), version 2.40.0	Python 3.12 component using the OpenAI Python SDK (openai), version 2.40.0

Note: "Not explicitly set" indicates that the parameter was omitted from the request. It does not imply the absence of a provider-imposed limit. The requested model identifier and the identifier returned in the response are reported separately to distinguish the requested model alias from the recorded response identifier.
