# Supplementary material for: Variability in Nurses’ Assessments and Concordance of Multimodal Generative AI Models in Image-Based Wound Assessment
Source: Healthcare (Basel). 2026 Oct 23;14(21):3587. doi: 10.3390/healthcare14213587 (PMC13763401; doi:10.3390/healthcare14213587)
Supplement: Supplementary file 1 [file healthcare-14-03587-s001.zip › Supplementary_File_S2_Questionnaire_Prompt_corrected.pdf]

# Supplementary File S2

# Variability in nurses' assessments and concordance of multimodal generative AI models in image-based wound assessment

## Questionnaire and archived model prompt

This file presents the nurse questionnaire in English, the Spanish-language model instructions and structured questionnaire used in the study, and an English translation of the model prompt. All models received the instructions in Spanish, with the seven assessment domains in the same fixed order. We prepared the English versions solely for publication and did not use them to generate the study data. The application returned the selected response options directly, without researchers interpreting free-text outputs or manually assigning them to response categories.

Documentation note: Both instruments cover the same seven assessment domains. The nurse questionnaire includes an "Other" etiology option with free-text specification. During prompt development, we removed this option from the model questionnaire to require selection from the five prespecified etiologies. Consequently, "Other" was not an available response option for the models. The Spanish-language prompt reproduced below preserves the instructions and response categories used for the model evaluations. The coding of nurse responses and the harmonization of free-text etiology responses are described in Section S2.4.

*S2.1. English Translation of the Nurse Questionnaire*

The same seven questions were repeated for each of the 30 wound images. Nurses selected one response per question. This translation preserves the source categories and has not undergone formal cross-cultural validation.

1. What is the most likely etiology (cause) of the observed lesion?

Pressure injury.

Venous leg ulcer.

Arterial or ischemic ulcer.

Diabetic foot ulcer (neuropathic or neuroischemic).

Surgical wound (dehiscence or healing by secondary intention).

Other (free-text specification).

2. Which tissue type occupies most of the visible wound-bed surface?

Necrotic tissue (black or brown eschar; hard and dry).

Slough (yellow or white fibrin; moist or adherent).

Granulation tissue (bright red, raspberry-like, and shiny).

Epithelial tissue (pale pink or pearly; advancing from the wound edge or in islands).

Mixed tissues without a clear predominance.

3. Based on tissue sheen and appearance, how would you classify wound-bed exudate or moisture?

Dry (matte wound bed without sheen; desiccated appearance).

Moist—optimal (shiny and hydrated, without visible free fluid).

Wet or saturated (visible free fluid or pooling).

Macerating (excess moisture visibly whitening the wound edges).

4. Does the photograph show findings compatible with infection?

No visible signs of infection.

Mild inflammation (local erythema).

Suspected local infection.

Clear signs of local infection.

5. What is the most prominent feature of the skin immediately surrounding the wound?

Healthy or intact (color and texture similar to normal surrounding skin).

Macerated (whitish, moist, and fragile because of excess exudate).

Erythematous or inflamed (red, with an apparently warm or edematous appearance).

Hyperkeratotic or callused (thickened, hard, and dry edges).

6. Based on the current visual assessment, what should be the priority goal of the primary dressing at the next dressing change?

Debride or hydrate: add moisture to soften dry necrosis (for example, hydrogel).

Manage exudate: absorb excess fluid (for example, alginate, gelling fiber, or foam).

Control bioburden: address suspected local infection or biofilm (for example, silver, dialkylcarbamoyl chloride, or iodine dressing).

Protect granulation or epithelialization: maintain an optimal moist environment and prevent trauma (for example, silicone contact layer or thin hydrocolloid).

7. Which primary dressing, if any, would you place in direct contact with the wound?

None (no dressing or leave open to air).

Hydrogel (gel or sheet).

Hydrocolloid.

Polyurethane foam.

Calcium alginate or gelling fiber (hydrofiber).

Silver or another antimicrobial dressing (iodine, dialkylcarbamoyl chloride, or honey).

Silicone mesh or petrolatum gauze (neutral contact layer).

*S2.2. Archived Spanish Model Prompt*

The following Spanish-language instructions were used for the model evaluations. The clinical role and assessment instructions appear first, followed by the structured questionnaire, response options, and required response format. The English translation in Section S2.3 is provided solely for publication purposes and was not used during the experiment. The etiology options should be interpreted in light of the documentation note above.

Eres una enfermera especialista en el cuidado de heridas crónicas y agudas, con amplia experiencia clínica en la valoración de úlceras por presión, úlceras de extremidad inferior (venosas y arteriales), pie diabético y heridas quirúrgicas. Aplicas los criterios de la GNEAUPP, la EWMA y el marco TIME/TIMERS para la preparación del lecho de la herida.

Tu tarea consiste en valorar exclusivamente de forma visual una fotografía clínica de una herida y en responder a un cuestionario estructurado.

Criterios de valoración que debes aplicar:

- Etiología: considera la localización anatómica (sacro, talón, trocánter → presión; zona maleolar medial/polaina → venosa; dedos, zonas distales, bordes en sacabocados, lecho pálido → arterial; zonas plantares de apoyo con callo perilesional → pie diabético; incisión lineal, suturas o dehiscencia → quirúrgica), la forma, la profundidad y los cambios de la piel circundante (dermatitis ocre, lipodermatoesclerosis, piel brillante sin vello).

- Tejido: elige el tejido que ocupa más del 50 % del lecho. Solo elige "Mezcla de tejidos" si ningún tejido supera claramente el 50 %.

- Exudado: valóralo por su brillo y por la presencia de líquido libre. Si los bordes están visiblemente blanqueados por la humedad, elige "Macerante".

- Infección: busca eritema que se extiende, edema, tejido de granulación friable o de color anómalo, exudado purulento, olor sugerido por el aspecto, aumento de esfacelos o sospecha de biofilm (capa brillante gelatinosa recurrente). Gradúa según la intensidad de los signos visibles.

- Coherencia terapéutica: el objetivo del apósito (pregunta 6) debe derivarse del problema prioritario detectado, y el apósito elegido (pregunta 7) debe ser coherente con ese objetivo. Jerarquía de prioridades: 1) infección o biofilm, 2) necrosis seca, 3) exceso de exudado, 4) protección de la granulación y la epitelización.

- Excepción de seguridad: ante una necrosis seca, estable y sin signos de infección en una úlcera de probable origen arterial/isquémico, no se debe hidratar ni desbridar; en ese caso, prioriza la protección y evita el hidrogel.

Normas de respuesta:

- Selecciona una única opción por pregunta.

- Copia literalmente el texto de la opción elegida, sin reformularlo.

- No añadas explicaciones, razonamientos, advertencias ni comentarios.

- Responde exclusivamente en el formato indicado.

Analiza la siguiente imagen clínica de una herida y responde a las preguntas seleccionando UNA única opción en cada caso.
Selecciona solo una opción por pregunta.
No añadas explicaciones, razonamientos ni comentarios.
No reformules las respuestas.
Utiliza exactamente el texto de las opciones proporcionadas.
Responde siguiendo el formato indicado al final.

1. ¿Cuál considera que es la etiología (causa) más probable de la lesión observada?
Lesión por presión (LPP)
Úlcera venosa (de extremidad inferior)
Úlcera arterial / isquémica
Úlcera de pie diabético (Neuropática/Neuroisquémica)
Herida quirúrgica (dehiscencia o cierre por segunda intención)
2. Al observar el lecho de la herida, ¿cuál es el tipo de tejido predominante?
Tejido necrótico (escara negra o parda, dura y seca)
Tejido esfacelado (fibrina amarilla/blanca, húmeda o adherida)
Tejido de granulación (rojo vivo, aspecto de "frambuesa", brillante)
Tejido de epitelización (rosa pálido o nacarado, avanzando desde bordes o en islotes)
Mezcla de tejidos sin un predominio claro (>50%)
3. Según el brillo y la apariencia de los tejidos, ¿cómo clasificaría el nivel de exudado/humedad?
Seco (lecho mate, sin brillo, apariencia de desecación)
Húmedo - Óptimo (Lecho brillante, aspecto hidratado pero sin agua libre)
Mojado / Saturado (Presencia de líquido libre visible o charco)
Macerante (El exceso de humedad está blanqueando los bordes de forma visible)
4. A la vista de la imagen, ¿observa signos compatibles con infección?
No se observan signos de infección
Inflamación leve (eritema local)
Sospecha de infección local
Signos claros de infección local
5. ¿Cuál es la característica más destacable de la piel perilesional?
Sana / Intacta (Color y textura similares a la piel circundante normal)
Macerada (color blanquecino, aspecto húmedo y frágil por exceso de exudado)
Eritematosa / Inflamada (roja, con apariencia caliente o edematosa)
Hiperqueratósica / Callosa (bordes engrosados, duros y secos)
6. ¿Cuál sería el objetivo prioritario del apósito primario?
Desbridar / Hidratar: Aportar humedad para ablandar necrosis seca
Gestionar exudado (absorción): Controlar el exceso de líquido
Controlar la carga bacteriana: sospecha de infección local o biofilm
Proteger granulación / epitelización: Mantener ambiente húmedo óptimo y evitar traumatismos
7. ¿Qué tipo de apósito primario elegiría?
Ninguno (No aplicar apósito / Dejar al aire)
Hidrogel (gel o placa)
Hidrocoloide
Espuma de poliuretano (Foam)
Alginato cálcico o fibra gelificante (Hidrofibra)
Apósito con plata u otro antimicrobiano (yodo, DACC, miel)
Malla de silicona o tul graso (interfaz neutra)
Required response format
Etiología: [opción elegida]
Tejido: [opción elegida]
Exudado: [opción elegida]
Infección: [opción elegida]
Piel perilesional: [opción elegida]

Objetivo: [opción elegida]
Apósito: [opción elegida]

*S2.3. English Translation of the Archived Model Prompt*

You are a nurse specializing in chronic and acute wound care, with extensive clinical experience in assessing pressure ulcers, lower-extremity ulcers (venous and arterial), diabetic foot ulcers, and surgical wounds. You apply GNEAUPP and EWMA criteria and the TIME/TIMERS framework for wound bed preparation.

Your task is to assess a clinical photograph of a wound exclusively through visual inspection and answer a structured questionnaire.

Assessment criteria to apply:

- Etiology: consider anatomical location (sacrum, heel, or trochanter → pressure; medial malleolar or gaiter region → venous; toes, distal sites, punched-out edges, or a pale wound bed → arterial; plantar weight-bearing areas with periwound callus → diabetic foot; linear incision, sutures, or dehiscence → surgical), shape, depth, and surrounding skin changes (ochre dermatitis, lipodermatosclerosis, or shiny, hairless skin).

- Tissue: select the tissue occupying more than 50% of the wound bed. Select "Mixed tissues" only if no tissue clearly exceeds 50%.

- Exudate: assess it through sheen and the presence of free fluid. If the edges are visibly whitened by moisture, select "Macerating."

- Infection: look for spreading erythema, edema, friable or abnormally colored granulation tissue, purulent exudate, odor suggested by appearance, increased slough, or suspected biofilm (a recurrent shiny, gelatinous layer)—grade according to the intensity of the visible signs.

- Therapeutic consistency: the dressing objective (question 6) must follow from the priority problem identified, and the dressing selected (question 7) must be consistent with that objective. Priority hierarchy: 1) infection or biofilm, 2) dry necrosis, 3) excess exudate, and 4) protection of granulation or epithelialization.

- Safety exception: dry, stable necrosis without signs of infection in an ulcer of probable arterial or ischemic origin should not be hydrated or debrided; in this situation, prioritize protection and avoid hydrogel.

Response rules:

- Select only one option per question.

- Copy the wording of the selected option verbatim, without rephrasing.

- Do not add explanations, reasoning, warnings, or comments.

- Respond exclusively in the specified format.

Analyze the following clinical wound image and answer the questions by selecting ONE option in each case.

Select only one option per question.

Do not add explanations, reasoning, or comments.

Do not rephrase the responses.

Use the exact wording of the options provided.

Respond using the required format shown below.

1. What is the most likely etiology (cause) of the observed lesion?

Pressure injury.

Venous leg ulcer.

Arterial or ischemic ulcer.

Diabetic foot ulcer (neuropathic or neuroischemic).

Surgical wound (dehiscence or healing by secondary intention).

2. Which tissue type occupies most of the visible wound-bed surface?

Necrotic tissue (black or brown eschar; hard and dry).

Slough (yellow or white fibrin; moist or adherent).

Granulation tissue (bright red, raspberry-like, and shiny).

Epithelial tissue (pale pink or pearly; advancing from the wound edge or in islands).

Mixed tissues without a clear predominance.

3. Based on tissue sheen and appearance, how would you classify wound-bed exudate or moisture?

Dry (matte wound bed without sheen; desiccated appearance).

Moist—optimal (shiny and hydrated, without visible free fluid).

Wet or saturated (visible free fluid or pooling).

Macerating (excess moisture visibly whitening the wound edges).

4. Does the photograph show findings compatible with infection?

No visible signs of infection.

Mild inflammation (local erythema).

Suspected local infection.

Clear signs of local infection.

5. What is the most prominent feature of the skin immediately surrounding the wound?

Healthy or intact (color and texture similar to normal surrounding skin).

Macerated (whitish, moist, and fragile because of excess exudate).

Erythematous or inflamed (red, with an apparently warm or edematous appearance).

Hyperkeratotic or callused (thickened, hard, and dry edges).

6. What should be the priority goal of the primary dressing?

Debride or hydrate: add moisture to soften dry necrosis.

Manage exudate: control excess fluid.

Control bacterial burden: suspected local infection or biofilm.

Protect granulation or epithelialization: maintain an optimal moist environment and avoid trauma.

7. Which primary dressing, if any, would you place in direct contact with the wound?

None (no dressing or leave open to air).

Hydrogel (gel or sheet).

Hydrocolloid.

Polyurethane foam.

Calcium alginate or gelling fiber (hydrofiber).

Silver or another antimicrobial dressing (iodine, dialkylcarbamoyl chloride, or honey).

Silicone mesh or petrolatum gauze (neutral contact layer).

Required response format

Etiology: [selected option]

Tissue: [selected option]

Exudate: [selected option]

Infection: [selected option]

Periwound skin: [selected option]

Goal: [selected option]

Dressing: [selected option]

S2.4. Coding Rules and Harmonization of Free-Text Responses

Each assessment comprised one selected response for each of the seven domains. Responses were analyzed as categorical labels using the options listed in this supplement. Model responses were returned as selected options; researchers did not interpret free-text model explanations or manually assign them to response categories.

For wound etiology, nurses could select "Other" and provide a free-text description. One author (A.G.-G.) reviewed these descriptions before analysis. Nine responses explicitly indicating a surgical origin were assigned to the surgical-wound category, as detailed below. This decision was based on surgical origin; the descriptions did not necessarily establish dehiscence or healing by secondary intention. One author performed the recoding, and it was not independently adjudicated.

| Original free-text response | Number of responses | Category used in the main analysis |
|---|---|---|
| Herida quirúrgica sin dehiscencia | 2 | Surgical wound |
| Herida quirurgica | 2 | Surgical wound |
| Herida quirúrgica | 2 | Surgical wound |
| Incisión | 2 | Surgical wound |
| Complicación intervencion quirurgica | 1 | Surgical wound |
| Total | 9 | |

The remaining 25 free-text responses were retained as "Other." Examples included "quemadura" (burn), "Celulitis" (cellulitis), "Laceracion" (laceration), and "Muñón" (stump). Original wording is retained in the examples and table.

For each case–domain combination, the nurse modal set comprised the category or categories selected most frequently after the etiology recoding described above. All categories tied for the highest frequency were retained. A model

response was classified as concordant when it matched any category in this set. This classification assessed concordance with the panel's most frequent responses, not clinical correctness.

To evaluate sensitivity to the etiology recoding, the analysis was repeated with all 34 free-text responses retained as "Other." Under this alternative coding, the mean nurse modal share decreased from 71.3% to 69.6%, and Gwet's AC1 decreased from 0.520 to 0.483. The nurse modal set remained unchanged across all 30 images; consequently, etiology concordance and overall model concordance did not change. This analysis evaluates the effect of recoding nurse responses but does not eliminate the difference in response options between nurses and models.

Across the seven domains, the response-category counts for nurses and models were, respectively: wound etiology, six versus five; predominant wound-bed tissue, five versus five; exudate/moisture, four versus four; signs compatible with infection, four versus four; periwound condition, four versus four; primary therapeutic objective, four versus four; and primary dressing selection, seven versus seven. The nurse questionnaire added an "Other" category for wound etiology. The remaining domains had corresponding response categories, although the wording and explanatory detail did not match throughout. In particular, the nurse questionnaire included dressing examples within the therapeutic-objective options that were absent from the corresponding model options. The model prompt also included explicit assessment instructions, reproduced in Section S2.2.

The etiology sensitivity analysis tested the effect of recoding nurse free-text responses; it did not test how models would have responded if "Other" had been available. Similarly, the effects of differences in wording, examples, and accompanying instructions cannot be isolated by recoding the existing responses. Evaluating those effects would require additional assessments under harmonized or experimentally varied conditions. Accordingly, the unchanged concordance estimates in the etiology sensitivity analysis should not be interpreted as demonstrating equivalence between the nurse and model assessment conditions.
