# Supplementary material for: Variability in Nurses’ Assessments and Concordance of Multimodal Generative AI Models in Image-Based Wound Assessment
Source: Healthcare (Basel). 2026 Oct 23;14(21):3587. doi: 10.3390/healthcare14213587 (PMC13763401; doi:10.3390/healthcare14213587)
Supplement: Supplementary file 1 [file healthcare-14-03587-s001.zip › Supplementary_File_S3_Reporting_Checklists_v2.pdf]

Supplementary File S3

Reporting checklists

Manuscript: Variability in nurses' assessments and concordance of multimodal generative AI models in image-based wound assessment.

This file maps the reporting of the study against the STROBE statement for cross-sectional studies, the Guidelines for Reporting Reliability and Agreement Studies (GRRAS), and the MI-CLEAR-LLM 2025 recommendations. Locations refer to sections, tables, and supplementary files. "Reported" indicates that the item is addressed at the stated location; "Not applicable" indicates that the item does not apply to the study design. Requirements are presented as concise paraphrases of the original checklist items.

S3.1 STROBE checklist for cross-sectional studies

Source: STROBE Initiative. STROBE checklist for cross-sectional studies. https://www.strobe-statement.org/checklists/. Items specific to cohort and case-control designs are omitted.

Item	Recommendation	Status	Location in manuscript and supplementary files
**Title and abstract**			
1a	Indicate the study design with a commonly used term in the title or abstract.	Reported	Abstract (Methods); Section 2.1. Exploratory cross-sectional comparative study.
1b	Provide an informative and balanced summary of what was done and found.	Reported	Abstract. Design, samples, agreement and concordance measures with 95% CIs, and the interpretive scope (no independent reference standard).
**Introduction**			
2	Explain the scientific background and rationale.	Reported	Section 1. Variability in wound assessment, purpose-built versus general-purpose AI, and the need to evaluate models against expert variability.
3	State specific objectives, including any prespecified hypotheses.	Reported	Section 1.1. General and specific objectives; exploratory design without prespecified hypotheses (Section 2.1).
**Methods**			
4	Present key elements of study design early in the paper.	Reported	Section 2.1. Same 30 images and seven domains assessed independently by 18 nurses and three models under image-only conditions.
5	Describe the setting, locations, and relevant dates.	Reported	Sections 2.2, 2.5 and 2.6. University hospital and primary care centres in northwestern Spain; image acquisition in May 2026; nurse and model evaluations in June 2026.
6a	Give eligibility criteria and the sources and methods of selection of participants.	Reported	Sections 2.3–2.4. Purposive quota selection of wounds (six per category) with exclusion criteria; purposive recruitment of nurses with explicit eligibility criteria.
7	Clearly define all outcomes and variables; give diagnostic criteria, if applicable.	Reported	Sections 2.5 and 2.7; Supplementary File S2. Seven categorical domains, modal share, nurse modal set, concordance and intermodel agreement. Diagnostic criteria do not apply because accuracy was not evaluated.

Item	Recommendation	Status	Location in manuscript and supplementary files
8	Give sources of data and details of methods of assessment; describe comparability across groups.	Reported	Sections 2.2, 2.5 and 2.6; Supplementary File S2. Standardized image acquisition, questionnaire development and content review, archived model prompt; nurses and models received the same images and domains in the same order, and the difference in the "Other" etiology option is disclosed.
9	Describe any efforts to address potential sources of bias.	Reported	Sections 2.2, 2.5, 2.6 and 4.1. Identical visual information, independent and blinded ratings, isolated model requests without follow-up prompts or manual editing, and sensitivity analyses for coding and ties.
10	Explain how the study size was arrived at.	Reported	Sections 2.3–2.4. Thirty cases fixed a priori by equal quota allocation, with methodological precedents; nurse panel size determined by participation (18/30). Precision is reported through confidence intervals.
11	Explain how quantitative variables were handled; describe groupings and why.	Reported	Sections 2.7–2.8. Modal shares analysed as continuous values; numerical strata (≥70%, 50% to <70%, <50%) used only for secondary description.
12a	Describe all statistical methods, including those used to control for confounding.	Reported	Section 2.8. Gwet's AC1, Fleiss' κ, case-clustered bootstrap, Wilson intervals, paired sign-permutation tests with Holm adjustment, and software. Confounder adjustment does not apply to this agreement design.
12b	Describe methods used to examine subgroups and interactions.	Reported	Sections 2.7–2.8. Domain-level analyses and secondary strata by nurse modal share.
12c	Explain how missing data were addressed.	Reported	Sections 2.4 and 2.6. Responses were mandatory; nurse characteristics and assessments were complete, and all model requests returned valid structured responses.
12d	Describe analytical methods taking account of the sampling strategy.	Reported	Section 2.8. The wound case is the sampling and resampling unit; case-clustered bootstrap preserves within-wound dependence, with the nurse panel treated as fixed.
12e	Describe any sensitivity analyses.	Reported	Section 2.8. Etiology recoding, single-mode rule for tied responses, and leave-one-nurse-out benchmark.
**Results**			
13a	Report numbers of individuals at each stage of the study.	Reported	Sections 2.3, 2.4 and 3. Patient selection until quotas were filled; 30 eligible nurses invited, 18 participated and completed all 30 cases; 30 images analysed.
13b	Give reasons for non-participation at each stage.	Reported	Section 2.4.
13c	Consider use of a flow diagram.	Reported	Considered; the single-stage recruitment is fully described in Sections 2.3–2.4 and 3, so a diagram was not required.
14a	Give characteristics of study participants.	Reported	Section 3, Table 1 (nurses); Supplementary File S1, Tables S4–S5 (wound categories and image characteristics). Patient data were not collected by design (Section 2.3).
14b	Indicate the number of participants with missing data for each variable.	Reported	Sections 2.4 and 3. No missing data for any variable.
15	Report numbers of outcome events or summary measures.	Reported	Section 3; Tables 2–4; Supplementary File S1, Tables S1–S6.

Item	Recommendation	Status	Location in manuscript and supplementary files
16a	Give unadjusted estimates and their precision.	Reported	Tables 2–4; Supplementary File S1, Tables S1–S3. Estimates with 95% CIs; paired differences with Holm-adjusted p values. Adjusted estimates do not apply.
16b	Report category boundaries when continuous variables were categorized.	Reported	Section 2.7 and Table 4 (≥70%, 50% to <70%, <50%).
16c	Translate relative risk into absolute risk, if relevant.	Not applicable	No risk estimates were calculated.
17	Report other analyses done.	Reported	Section 3; Table 4; Supplementary File S1, Tables S1–S3 and S6.
**Discussion**			
18	Summarise key results with reference to study objectives.	Reported	Section 4 (first paragraph) and Section 5.
19	Discuss limitations, including sources of potential bias or imprecision.	Reported	Section 4.1.
20	Give a cautious overall interpretation of results.	Reported	Section 4. Agreement, concordance, accuracy and clinical validity are distinguished; findings are compared with previous studies; exploratory comparisons are identified.
21	Discuss the generalisability of the study results.	Reported	Sections 4.1–4.2.
**Other information**			
22	Give the source of funding and the role of the funders.	Reported	Funding statement. No external funding.

S3.2 GRRAS checklist

Source: Kottner J, et al. Guidelines for Reporting Reliability and Agreement Studies (GRRAS) were proposed. Int J Nurs Stud. 2011;48:661–671. https://doi.org/10.1016/j.ijnurstu.2011.01.016.

Item	Checklist item	Status	Location in manuscript and supplementary files
**Title and abstract**			
1	Identify in the title or abstract that interrater/intrarater reliability or agreement was investigated.	Reported	Title; Abstract; Section 2.1. Inter-nurse agreement and model concordance.
**Introduction**			
2	Name and describe the diagnostic or measurement device of interest.	Reported	Sections 2.5–2.6; Supplementary File S2. Seven-domain image-based wound-assessment questionnaire; three named multimodal generative AI models.
3	Specify the subject population of interest.	Reported	Sections 2.2–2.3. Thirty wounds, one per patient, across five wound categories.
4	Specify the rater population of interest.	Reported	Sections 2.4 and 2.6. Registered nurses with wound-care expertise; three commercial models.
5	Describe what is already known about reliability and agreement and provide a rationale.	Reported	Section 1. Previous evidence of inter-clinician variability and rationale for contextualizing model concordance.
**Methods**			

Item	Checklist item	Status	Location in manuscript and supplementary files
6	Explain how the sample size was chosen; state the number of raters, subjects and replicate observations.	Reported	Sections 2.3, 2.4 and 2.6. Thirty cases fixed a priori; 18 nurses; three models; one assessment per rater and image.
7	Describe the sampling method.	Reported	Sections 2.3–2.4. Purposive quota sampling of wounds; purposive sampling of nurses.
8	Describe the measurement/rating process.	Reported	Sections 2.2, 2.5 and 2.6. Standardized photographs, no clinical information, fixed case sequence, single isolated model requests; no repeated measurements.
9	State whether measurements/ratings were conducted independently.	Reported	Sections 2.5–2.6. Nurses were blinded to other ratings and AI outputs; model requests excluded prior or other evaluators' responses.
10	Describe the statistical analysis.	Reported	Sections 2.7–2.8. Modal shares, Gwet's AC1, Fleiss' κ, concordance, case-clustered bootstrap, Wilson intervals, paired comparisons and sensitivity analyses.
**Results**			
11	State the actual number of raters and subjects and the number of replicate observations.	Reported	Section 3; Tables 1 and 3. Eighteen nurses and three models assessed 30 wounds across seven domains; one assessment per rater and image.
12	Describe the sample characteristics of raters and subjects.	Reported	Table 1 (nurse age, sex, experience, setting and education); Supplementary File S1, Tables S4–S5 (wound categories and image characteristics).
13	Report estimates of reliability and agreement, including measures of statistical uncertainty.	Reported	Tables 2–4; Supplementary File S1, Tables S1–S3 and S6. All estimates with 95% CIs.
**Discussion**			
14	Discuss the practical relevance of results.	Reported	Sections 4–5.
**Auxiliary material**			
15	Provide detailed results, if possible.	Reported	Supplementary Files S1 (Tables S1–S6) and S2 (questionnaire, prompt and coding rules); Data Availability Statement.

S3.3 MI-CLEAR-LLM 2025 checklist

Source: Park SH, et al. Minimum Reporting Items for Clear Evaluation of Accuracy Reports of Large Language Models in Healthcare (MI-CLEAR-LLM): 2025 Updates. Korean J Radiol. 2025;26:1123–1132. https://doi.org/10.3348/kjr.2025.1522. The recommendations apply to reporting model use; the study evaluates concordance with a nurse-derived comparator rather than diagnostic accuracy.

Item	Checklist item	Status	Location in manuscript and supplementary files
**1. Model identification**			
1a	Model name, version and developer.	Reported	Section 2.6. GPT-5 (OpenAI), Gemini 2.5 Flash (Google) and Claude Sonnet 4.6 (Anthropic), with API identifiers and versions.
1b	Proprietary or open-source status.	Reported	Section 2.6. All three models are proprietary.
1c	Date of access and query execution.	Reported	Section 2.6. Exact query dates in June 2026.
1d	Modifications, weight source and release tag for self-managed models; model sharing.	Not applicable	Proprietary models accessed through APIs; no self-managed deployment.

Item	Checklist item	Status	Location in manuscript and supplementary files
1e	Training-data cutoff date, when known.	Reported	Section 2.6. Developer-reported cutoffs, all preceding image acquisition.
**2. Access mode**			
2a	Access method.	Reported	Section 2.6. API access through an investigator-developed web application.
2b	Rationale for the chosen access mode.	Reported	Section 2.6.
2c	System-level features beyond the LLM (e.g., system prompts, memory).	Reported	Section 2.6. Placement of instructions, stateless requests without memory, and absence of external tools.
2d	Computational environment for local deployment.	Not applicable	No local deployment.
**3. Input data type**			
3	Type and format of input data sufficient for replication.	Reported	Sections 2.2 and 2.6; Supplementary File S2. One deidentified photograph per request (file format and resolution, Base64 encoding without resizing) plus the Spanish text prompt; no clinical data.
**4. Adaptation strategy**			
4a	Adaptation method and whether model weights were altered.	Reported	Section 2.6. No fine-tuning or retrieval augmentation; adaptation limited to prompt design.
4b	Precise terminology for non-parametric adaptation.	Reported	Section 2.6. “Prompt development” is distinguished from model training.
4c	Detailed description of the adaptation process.	Reported	Section 2.6; Supplementary File S2.
**5. Prompt optimization**			
5a	Steps taken to create the prompts and rationale for wording.	Reported	Section 2.6. Prompt aligned with the nurse questionnaire and GNEAUPP, EWMA and TIME/TIMERS guidance; rationale for removing “Other”.
5b	Prompting strategies used.	Reported	Section 2.6. Role prompting, explicit assessment rules and priority hierarchy, zero-shot, constrained structured output without requested reasoning.
5c	Full, directly executable prompt text.	Reported	Supplementary File S2, Sections S2.2 (archived Spanish prompt) and S2.3 (English translation).
5d	Summary of the prompt optimization process.	Reported	Section 2.6. Number of iterations and nature of interim changes; no study images used during development.
**6. Prompt execution**			
6a	Query submission configuration.	Reported	Section 2.6. One independent API request per image and model, application reset between requests, no prior exchanges or follow-up prompts.
6b	Experiment script, if feasible.	Reported	Data Availability Statement. Application source code and analysis code available on request.
**7. Stochasticity**			
7a	Technical parameters affecting randomness.	Reported	Section 2.6. Generation settings reported.
7b	Number of querying attempts.	Reported	Section 2.6. One request per model and image.
7c	Method for synthesizing multiple responses.	Not applicable	A single response was obtained per model and image.

Item	Checklist item	Status	Location in manuscript and supplementary files
7d	Reliability of outputs across attempts.	Not applicable	Not applicable to single-attempt evaluation; addressed as a direction for future research in Section 4.1.
**8. Test data independence**			
8a	Overlap between test data and training or adaptation data.	Reported	Sections 2.2 and 2.6. Images acquired in May 2026, after all training-data cutoffs, never published, and not used for prompt development.
8b	Nature and source of adaptation and test data.	Reported	Sections 2.2–2.3 and 2.6.
8c	URLs and prior exposure of test data sourced online.	Not applicable	Images were acquired locally and are not publicly available.

AI, artificial intelligence; API, application programming interface; CI, confidence interval; LLM, large language model.
